# HIV Post Exposure Prophylaxis and Risk of Relapse in Adolescent With Bipolar Illness and Psychopharmacologic Challenges

**DOI:** 10.7759/cureus.13777

**Published:** 2021-03-09

**Authors:** Mayank Gupta

**Affiliations:** 1 Psychiatry, Lake Erie College of Osteopathic Medicine, Erie, USA; 2 Psychiatry and Behavioral Sciences, Clarion Psychiatric Center, Clarion, USA

**Keywords:** bipolar, hiv, post exposure prophylaxis, pep, adolescents, truvada, raltegravir

## Abstract

Bipolar disorder (BD) in adolescents is associated with risky behaviors, including high risk for sexually transmitted infections. When exposed, post exposure prophylaxis (PEP) is recommended within 72 hours for a period of 28 days. The medications used for PEP are known to have common neuropsychiatric side effects, renal toxicity and risk of hepatic injury. The concomitant use of PEP and bipolar medications may have serious additive adverse effects which needs careful assessment and monitoring. PEP medications, in particular raltegravir, are known to have a common side effect of insomnia. The medication options may be more limited during this period and since insomnia is also known to precipitate mania it needs to be addressed. The knowledge of these side effects of PEP medications, understanding its interactions with mood stabilizers like lithium and valproic acid is important when caring for these individuals. The medication options of monotherapy or combination regimen for BD must be discussed with the patient and informed choices would yield better clinical outcomes. Although there is no established standard, but weekly monitoring of complete blood counts and liver functions until PEP is completed would be highly recommended to prevent serious negative events.

## Introduction

Bipolar disorder (BD) in children and adolescents has an estimated prevalence of approximately 1.8% [1]. BD is associated with increased risk of sexually transmitted infections (STI) and in particular human immunodeficiency virus (HIV) with hazard ratio of 3.59 [2]. According to National Epidemiologic Survey of Alcohol and Related Disorders it was found that the 12-month prevalence of BD among HIV-infected respondents was 10.8%, compared to 6.2 among HIV-negative respondents [3]. Behavioral disinhibition, impaired judgement and hypersexuality [4] have been widely reported in child and adolescent population with BD [5]. The Centers for Disease Control and Prevention (CDC) has recommended post exposure prophylaxis (PEP) within 72 hours of unprotected sex with unknown partner or/and after sexual assault. PEP normally consists of three anti-HIV drugs, from two of the different classes. The most recent guidelines recommend using Truvada (a fixed-dose combination tablet combining emtricitabine and tenofovir) from the nucleoside/nucleotide reverse transcriptase inhibitors (NRTI class), and raltegravir (Isentress) from the integrase inhibitor class. There are alternative treatment options available. There are few major drug interactions of these medications with pharmacologic agents used for bipolar disorder. Tenofovir disoproxil fumarate (TDF) and lithium are associated with renal tubular toxicity, which could be additive, or a pharmacokinetic interaction may occur at renal transporters with a decrease in TDF elimination. Truvada is also associated with mild transaminitis and in few reports efavirenz leads to hepatotoxicity which has been associated with need for transplantation and also death. In last five years, there has been extensive empirical evidence of neuropsychiatric side effects of raltegravir (RAL) [6]. The most common side effect of RAL is reported as an acute onset insomnia [7]. The number of these cases and presentations are rising and often lead to serious clinical issues. Therefore, this case report provides much needed insight to understand the nature and severity in real world clinical settings.

## Case presentation

The patient is a 17-year-old male who initially presented to the Emergency Department (ED) with his family after he was sexually assaulted at the high school event. He underwent complete necessary medical workup including Sexual Assault Evidence Kit (SAEK). The patient had flight of ideas, grandiosity and marked disinhibition suggestive of underlying bipolar illness. Patient's family reported he was nonadherent with medications for two weeks prior to this event. There is a strong family history of bipolar in father side of the family. The patient has been diagnosed with bipolar I disorder since age 16 and had three prior inpatient hospitalization. He has a history of non-adherence with treatment, with previous trails of aripiprazole, quetiapine and valproic acid. After the last discharge from the mental health institution, he was stabilized on lithium carbonate and olanzapine combination. The patient stopped his lithium carbonate and olanzapine, and the family found medication hidden at home. He was sexually assaulted at a party and found on the street under the influence of alcohol which led to this admission. He was also found positive for cannabis on urine toxicology. He was restarted on the combination of lithium 300mg two times a day and olanzapine 10mg daily. He had partial response within five days of this therapy. Legal guardians also consented for PEP recommended by the medical team but they withdrew consent for lithium due to the risk of renal toxicity related to the interaction with Truvada. The patient, on day 5 of starting raltegravir, developed a new onset insomnia. At day 10, he again started to develop mania with symptoms of marked disinhibition, flight of ideas, insomnia and grandiosity. Patient’s family reported that he never had similar episode even without the medications. The medical team also ruled out organic cause of mania. The patient denied use of any other substances which are not usually found on urine toxicology. Patient’s olanzapine was increased to 20mg over a period of three days with little improvement. He developed mild transaminitis on day 14 which progressed on day 18. Subsequently, olanzapine was tapered off and asenapine was initiated at 5mg and then titrated to 20mg in divided doses over five days. The patient did report significant insomnia which responded poorly to melatonin, trazodone but partially responded to 5mg of zolpidem. Based on clinical discussion, this new onset insomnia was possibly attributed to raltegravir. After the completion of PEP on day 28, the patient started to demonstrate clinical improvement on asenapine. His sleep was improved and reduction in manic symptoms was evident on day 33. Later, lithium was also restarted and titrated to 450mg two times daily with therapeutic level of 0.8. Patient's liver functions improved and he was discharged in the care of family on day 40 to start partial hospital program.

## Discussion

Lack of insight, non-adherence with medications, diagnostic ambiguity [8], cheeking of medications, stigma about medications [9], lowering of risk perception of cannabis [10-11] since legalization, patient’s assent and informed parental consents are common impediments to effectively treat juvenile bipolar mania on inpatient settings. In these circumstances, astute clinician would juxtapose various psychotherapeutic interventions to address these barriers [12-13]. However, amid these challenges it is imperative to know the serious interactions of HIV PEP medications with psychopharmacologic interventions of BD. PEP medications also have their own neuropsychiatric side effects. A well-informed provider may offer more informed choices and thereby develop trust and rapport with both patient and families. The treatment of bipolar mania is challenging when choice of medications is limited in patients receiving PEP. The use of lithium carbonate with tenofovir disoproxil fumarate (TDF) is associated with risk of renal toxicity [14]. Truvada is also associated with mild transaminitis, which could be accentuated with use of valproic acid, carbamazepine and oxcarbamazepine. The atypical antipsychotics are mainstay for the treatment in these circumstances, although paliperidone is known to have minimal cytochrome P450 interaction and less hepatic metabolism but often leads to elevated prolactin levels [15]. The males do not prefer this medication due to the risk of gynecomastia and females avoid it due to risk of galactorrhea. Lurasidone is not effective for mania episodes. Aripiprazole and quetiapine were also effective options but given the additive effects of PEP medications, we recommended weekly complete blood counts and liver function tests to monitor neutrophils counts and signs of liver toxicity. The most concerning side effect of PEP medications is insomnia which in itself is known to precipitate mania in vulnerable population [16]. It remains unclear if insomnia is related to mania or accentuated by a medication side effect. However, careful history and collateral information from families and establishing a timeline of onset of these symptoms could provide useful insights. If patient does not respond to melatonin, hydroxyzine or trazodone then off label use of zolpidem may be an option. In summary, psychopharmacologic options could be limited until the PEP is completed, therefore informed consent to pursue monotherapy with antipsychotics only regimen or combination regimen including mood stabilizer must be discussed. The clinicians must address insomnia with both pharmacologic and non-pharmacologic interventions. During this period, careful monitoring of the patient’s mental status, patient psychoeducation, weekly blood tests monitoring, and family meetings would yield better clinical outcomes.

## Conclusions

The treatment of bipolar mania with concomitant HIV PEP limits options for effective medication management. Patients with BD remain at high risk of STI, therefore understanding the pharmacologic profile these PEP agents have is important for using evidence-based interventions. A collaborative approach with pediatricians and infectious diseases experts would be ideal when available; however, in rural settings knowledge of the side effects profile and drug interaction would avoid negative outcomes. A well-informed clinician may anticipate a clinical course during PEP which usually lasts for 28 days. When patients are educated, and families are informed about plan to address this unique clinical condition, a better overall clinical outcome could be expected.
